# What Are the Potential Therapeutic Benefits of Targeting Blood-Borne Lipoproteins in the Treatment of Alzheimer’s Disease?

**DOI:** 10.3390/cells15131191

**Published:** 2026-06-30

**Authors:** Jérôme Robert

**Affiliations:** Institute for Clinical Chemistry, University Hospital of Zurich, University of Zurich, Raemistrasse 100, 8091 Zurich, Switzerland; jerome.robert@usz.ch or jerome.robert@uzh.ch

**Keywords:** HDL, LDL, Lp(a), Alzheimer’s disease, VLDL, CETP, statin, ANGPTL, PCSK9

## Abstract

Alzheimer’s disease (AD) is a progressive neurodegenerative disorder characterized by cognitive decline, the deposition of amyloid-β (Aβ) plaques, the formation of neurofibrillary tangles, and cerebrovascular dysfunction. Evidence suggests that blood-borne lipoproteins play a role in the disease’s pathophysiology by influencing the cerebrovasculature and amyloid metabolism. Low-density lipoprotein (LDL) and very-low-density lipoprotein (VLDL) can contribute to oxidative stress, endothelial dysfunction, vascular dysfunction, and the accumulation of amyloidogenic peptides, thereby exacerbating neurodegeneration. The role of lipoprotein(a) (Lp(a)) remains unclear, whereas high-density lipoprotein (HDL) is recognized for its cerebroprotective properties, including anti-inflammatory and vasoreactive functions. These properties help to maintain neuronal homeostasis and facilitate the clearance of Aβ from the brain. This review summarizes the current evidence regarding the role of lipoproteins in AD and discusses how therapeutic strategies targeting lipoprotein pathways, such as lipid-lowering agents and HDL mimetics developed for cardiovascular diseases, may benefit patients with AD.

## 1. Introduction

Neurodegenerative diseases, including Alzheimer’s disease (AD), represent a growing global health challenge [1]. While AD pathophysiology involves multiple complex mechanisms, accumulating evidence implicates dysregulated lipid metabolism as a critical contributor [2]. Cholesterol, one of the most abundant lipids within the brain, is essential for maintaining the integrity of neuronal membranes, forming synapses, and releasing neurotransmitters [3]. However, aberrant cholesterol homeostasis has been linked to neuroinflammation, amyloid-beta (Aβ) accumulation, and neuronal dysfunction [2]. Notably, cholesterol metabolism in the central nervous system (CNS) is largely segregated from the periphery because the blood–brain barrier (BBB) effectively prevents significant cholesterol exchange between circulation and the brain [4]. Despite this segregation, accumulating evidence suggests that plasma cholesterol levels are associated with the risk of neurodegenerative diseases. Interestingly, this relationship appears to depend not only on the absolute concentration of cholesterol but also on the specific lipoprotein carriers through which cholesterol is transported in the bloodstream.

**Figure 1:**
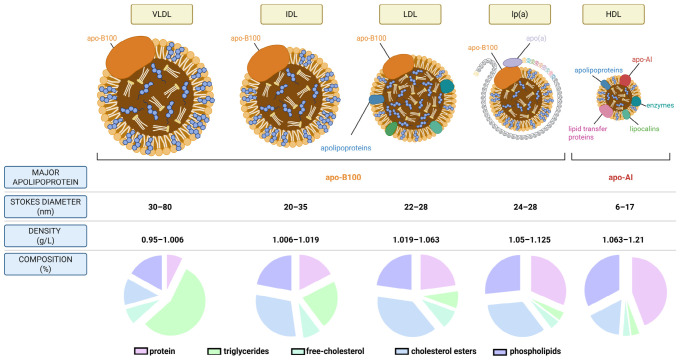
**Blood-borne lipoprotein characteristics.** Several classes of lipoprotein exist in the blood, varying in their content of cholesterols (esters, and free), phospholipids, triglycerides, and proteins. The respective concentrations of each are depicted at the bottom of the figure. apo = apolipoprotein, HDL = high-density lipoprotein, IDL= intermediate-density lipoprotein, LDL = low-density lipoprotein, Lp(a) = lipoprotein (a), VLDL = very-low-density lipoprotein [8,9,10]. BioRender.com. License Robert J. (2026).

In plasma, cholesterol is transported by lipoproteins, with low-density lipoprotein (LDL) being the primary carrier (Figure 1). LDL particles are enriched in cholesterol esters and contain a single molecule of apolipoprotein B (apoB), which serves as a structural protein and ligand for cellular uptake. LDL has been extensively studied in the context of atherosclerotic cardiovascular disease (ASCVD) [5]. In contrast, high-density lipoprotein (HDL), which contains apoA-I, is involved in reverse cholesterol transport, the process by which cholesterol is brought back from the periphery to the liver for excretion [6]. Emerging evidence suggests that these distinct lipoprotein classes may influence neurodegenerative processes differently. LDL may contribute to vascular dysfunction and inflammation, whereas HDL particles may exert protective effects through anti-inflammatory and amyloid-clearing properties [7]. Together, these observations highlight the critical role of blood-borne lipoproteins in the neurodegenerative disease risk and their potential as therapeutic targets.

This review examines the associations between blood-borne lipoproteins and AD, as well as the effects of lipid-lowering medications and HDL mimetics on neurodegenerative disease outcomes.

## 2. Apolipoprotein B-Containing Lipoproteins and Alzheimer’s Disease 

In the liver, apoB is assembled with lipids to form very-low-density lipoprotein (VLDL), which consists of relatively large particles (~30–80 nm in diameter) released into the circulation. As these particles deliver triglycerides to peripheral tissues, they are progressively hydrolyzed by lipoprotein lipase (LPL), endothelial lipase (EL), and hepatic lipase (HL). This process shrinks the particles and causes them to lose triglycerides to form intermediate-density lipoprotein (IDL, ~20–35 nm in diameter) and, ultimately, smaller, denser LDL particles (~24–28 nm in diameter) (Figure 1). These cholesterol-enriched LDL particles retain apoB as their main structural protein and serve as the primary carriers of cholesterol in the bloodstream [8]. While LDL cholesterol (LDL-C) reflects the cholesterol content within LDL particles, apoB represents the total number of atherogenic lipoprotein particles. Preclinical models, epidemiological studies, genetic analyses, and randomized controlled trials consistently demonstrate that higher levels of LDL-C and apoB are directly linked to an increased incidence of major adverse cardiovascular events [5]. This supports their use as biomarkers and therapeutic targets in cardiovascular disease prevention. However, the role of LDL—the levels of cholesterol and apoB—in neurodegenerative diseases is less direct and more complex than in cardiovascular pathology.

Mendelian randomization studies have investigated the causal relationship between genetically predicted LDL-C levels and AD risk. One such analysis, which incorporated genetic variants associated with LDL-C, found that variants that lower LDL-C through proprotein convertase subtilisin/kexin type 9 (*PCSK9*)—a gene coding for a serine protease that regulates cholesterol homeostasis by promoting LDL receptor (LDLR) degradation—or 3-hydroxy-3-methylglutaryl-coenzyme A reductase (*HMGCR*)—a gene coding for the primary rate-limiting enzyme in the mevalonate pathway that produces cholesterol—had inconsistent effects on AD risk [11]. In contrast, a separate Mendelian randomization study analyzing 144 single-nucleotide polymorphisms (SNPs) in various genes altering cholesterol levels revealed that a genetically predicted high LDL-C level was positively associated with an increased risk of both sporadic and familial AD [12]. A recent systematic review and meta-analysis of Mendelian randomization studies further supports a potential causal association between high genetically predicted LDL-C levels and an increased AD risk in both fixed-effects and random-effects models [13]. 

Early population-based studies have reported inconsistent associations between LDL-C levels and dementia risk. For example, data from the Atherosclerosis Risk in Communities (ARIC) study, which included more than 10,000 participants, demonstrated that LDL-C concentrations below 2.6 mmol/L were associated with a slower 20-year decline in executive function and attention [14]. Similarly, in the Three-City Study (3C Study) in France, which followed 7470 participants over the age of 65 for up to 13 years, elevated levels of LDL-C (≥4.25 mmol/L for women and ≥4.00 mmol/L for men) were associated with an increased risk of AD and all types of dementia, but not vascular dementia [15]. In contrast, findings from cohorts of Northern Manhattan residents, each with over 1000 participants, did not identify a significant association between LDL-C concentrations (including thresholds below 4.1 mmol/L) and cognitive performance over approximately seven years of follow-up [16]. Finally, a Manhattan cohort-based study with 1130 individuals over 65 years old who were followed for 18 months found that levels of LDL-C above 3.1 mmol/L were associated with a decreased risk of AD. Longer follow-up did not change this relation [17]. These discrepancies likely reflect the small sample sizes and methodological heterogeneity of the studies, including LDL-C categorization, the follow-up duration, and population characteristics such as age, ethnicity, education, and sex. Recent large-scale epidemiological studies have demonstrated an association between higher LDL-C levels and an increased risk of dementia. However, the strength and direction of this association vary by the age at measurement and follow-up duration. For example, in a cohort of 217,960 participants across eleven Korean hospitals followed over a period of 34 years, individuals with an LDL-C level below 1.8 mmol/L were significantly less likely to develop AD or any type of dementia than individuals with an LDL-C level above 3.4 mmol/L. Interestingly, a very low level of LDL-C (<0.8 mmol/L) did not reduce the risk any further, suggesting a threshold effect for optimal cognitive benefit [18]. Meanwhile, another Korean study conducted over 8 years reported that very low levels of LDL-C in individuals over 40 years old increased the risk of all-cause dementia in a U-shaped relationship [19]. Similarly, a retrospective cohort study of 1,853,954 individuals found that higher LDL-C was associated with a modest increase in dementia risk, with an adjusted rate ratio of 1.05 (95% CI, 1.03–1.06) per standard deviation increase [20]. The strongest association was observed in individuals younger than 65 years at baseline, particularly for dementia diagnosed more than 10 years later (RR: 1.17; 95% CI: 1.08–1.27), suggesting that midlife LDL-C levels may be a more relevant, modifiable risk factor than late-life levels [20]. Conversely, in very old adults, this relationship may be reversed or attenuated. A meta-analysis of individual participant data from over 21,000 individuals aged 60 years and older found no significant relationship between LDL-C levels and incident dementia or cognitive decline (OR: 1.01; 95% CI: 0.89–1.13) [21]. This age-related divergence may reflect survivor bias, competing mortality risks, or genuine biological differences. 

In addition to LDL-C, a UK Biobank (UKB) cohort study of 469,466 participants found that higher levels of apoB—the primary apolipoprotein in LDL particles—were associated with an increased risk of dementia (HR: 1.12, 95% CI 1.01–1.24 for the highest quartile), while higher apoB/apoA-I—the major apolipoprotein of HDL—ratios showed an even stronger association (HR: 1.23, 95% CI 1.11–1.37 per standard deviation increase) [22]. Novel findings in a cross-sectional study of 22 patients with mild cognitive impairment associate electronegative LDL-C subfractions—a modified, pro-atherogenic subfraction of LDL—with AD. In particular, the levels of L5, which is the most electronegative subfraction, were mildly yet significantly negatively correlated with cognitive performance (r = −0.431, *p* = 0.045), especially in the domains of orientation and language [23]. These results suggest that qualitative aspects of LDL particles (size, charge, oxidation, lipid and protein composition, etc.), beyond the total LDL-C concentration, may contribute to cognitive impairment. However, these observations must be repeated in larger cohorts. In addition to its potential role in cognitive decline, direct neuropathological evidence links LDL-C to AD pathology. A longitudinal clinical–pathological study of 559 participants found that higher premorbid LDL-C was associated with an increased burden of AD neuropathology, independently of the *APOE* genotype, which is the major genetic risk factor for sporadic AD [24]. Elevated LDL-C was associated with a greater neurofibrillary tangle burden (standardized mean difference [SMD]: 3.2; 95% CI, 1.2–5.2), beta-amyloid deposition (SMD, 2.9; 95% CI, 1.0–4.8), higher Braak scores (SMD: 2.6; 95% CI, 0.6–4.7), and increased cerebral amyloid angiopathy (CAA), which corresponds to the deposition of Aβ within the cerebrovasculature (SMD: 3.5; 95% CI, 0.9–6.0). Altogether, these findings support the hypothesis that atherogenic LDL particles may contribute to dementia pathogenesis, potentially via action on the cerebrovasculature. This is particularly true given that apoB is largely absent from the brain and does not cross the BBB or the blood–cerebrospinal fluid barrier [25]. The very small amount of apoB in the cerebrospinal fluid (CSF) is believed to originate from microglia, where messenger RNA is detected [26]. Notably, the level of apoB in CSF is strongly associated with early tau dysregulation in asymptomatic individuals and helps to identify those at risk of developing cognitive decline over time [26]. 

In addition to LDL, numerous studies have shown that the midlife levels of triglycerides, which are mostly transported via VLDL, are positively associated with an increased risk of cognitive impairment later in life. However, the results vary depending on the demographics of the population studied [27]. For instance, a large Danish study of 125,727 participants revealed that individuals in the 50th percentile for non-fasting triglycerides (median ~1.00 mmol/L) experienced a reduced risk of dementia and stroke compared to individuals with higher levels [27]. Conversely, in a diverse U.S. cohort of 16,170 participants, elevated fasting triglycerides (≥1.69 mmol/L) were associated with cognitive impairment exclusively among white women [28]. Preclinical and biomarker studies further suggest that midlife hypertriglyceridemia may facilitate the accumulation of toxic amyloid species in the brain. Longitudinal data indicate that individuals with high triglyceride levels in midlife are more likely to exhibit tau pathologies and brain amyloid deposition two decades later [29]. This highlights a potential mechanistic link between VLDL-mediated triglyceride delivery, LDL formation, and AD. Interestingly, in older adults (over 65 years old), mildly elevated triglyceride levels may be associated with a lower risk of cognitive decline [30]. This suggests that the relationship between triglyceride levels and the risk of developing dementia changes over the course of a person’s lifetime.

Taken together, these studies suggest a role for apoB-containing lipoproteins in AD. However, it is unclear whether this effect is direct or an indirect consequence of reduced cardiovascular risk. Further preclinical and in vitro research is therefore necessary to determine the precise relationship between AD and apoB lipoproteins. Notably, apoB100 overexpression in mice promotes key pathological features, including elevated LDL and reduced HDL levels, leading to increased lipid peroxidation, Aβ accumulation, and cerebrovascular apoB deposition [31]. Despite these pronounced pathological changes, double-transgenic apoBxAPP mice did not exhibit greater cognitive deficits than APP-only mice, highlighting a disconnect between the pathology and measurable behavioral impairment. 

## 3. Lipoprotein(a) and Alzheimer’s Disease

Lipoprotein(a) (Lp(a)) is an LDL-like particle composed of apoB covalently bound to apolipoprotein(a), a highly polymorphic glycoprotein characterized by variable numbers of kringle IV type 2 (KIV-2) repeats [9]. The level of plasma Lp(a) is largely genetically determined and remains relatively stable throughout life. Physiologically, Lp(a) may contribute to wound healing and tissue repair, but elevated concentrations above 125 nmol/L are strongly associated with ASCVD [9]. Lp(a) promotes cardiovascular risk through several mechanisms, including the deposition of cholesterol within the arterial walls, the enhancement of vascular inflammation, and interference with fibrinolysis due to the structural similarity of apolipoprotein(a) (apo(a)) to plasminogen [32]. High Lp(a) levels are independently associated with increased risks of coronary artery disease, ischemic stroke, peripheral arterial disease, and calcific aortic valve stenosis, making it an important inherited cardiovascular risk factor [32]. While its cardiovascular effects are well characterized, the relationship between Lp(a) and neurodegenerative diseases, particularly AD, remains complex and paradoxical. Unlike its established pro-atherogenic role in cardiovascular disease, accumulating evidence suggests that Lp(a) may exert protective effects against AD, challenging the conventional assumption about apoB lipoprotein-mediated neurodegeneration.

Mendelian randomization studies have provided genetic evidence supporting a protective role for Lp(a). An analysis of 54,162 individuals of European ancestry demonstrated that genetically predicted increases in Lp(a) were associated with a reduced risk of AD, with an odds ratio of 0.94 (95% CI 0.91–0.97, *p* = <0.001) [33]. The protective association is further supported by a prospective cohort study by Kunutsor and colleagues, which followed 2532 middle-aged men for a median of 24.9 years [34]. Men in the highest quartile of Lp(a) levels compared to the lowest quartile exhibited a hazard ratio of 0.68 (95% CI 0.47–0.99, *p* = 0.044) for dementia in an age-adjusted analysis, which persisted after adjustment for multiple cardiovascular risk factors, lipid parameters, and socioeconomic status (HR 0.68, 95% CI 0.46–0.99, *p* = 0.044) [34]. Notably, this protective effect was attenuated when controlling for mortality, suggesting that the apparent benefit may partly reflect survival bias or competing risks. Larsson et al. used a Mendelian randomization approach with 367,586 European-descent participants from the UKB and found null associations between genetically predicted Lp(a) levels and AD or vascular dementia, likely due to limited statistical power [35]. However, they uncovered a weak inverse association with a self-reported parental history of AD or dementia, suggesting that higher Lp(a) levels may provide some protection. Inversely, in a cohort study of 254,575 women and 214,891 men from the UKB, Gong et al. found no evidence of an association between Lp(a) levels and dementia risk. Compared with the lowest quartile, the HR for the highest quartile was 0.96 (95% CI 0.87–1.07) [22]. Finally, Thomas and colleagues recently examined three large cohort studies with 539,478 individuals from the Copenhagen General Population Study (CGPS), the Copenhagen City Heart Study (CCHS), and the UKB, with follow-up extending to 30.2 years [36]. During this period, 6404 cases of AD and 7866 cases of vascular-related dementia were documented. In the Copenhagen and UKB cohorts, the plasma level of Lp(a) was not associated with the risk of AD, vascular-related dementia, or all-cause dementia on continuous scales. However, after accounting for competing risks, the absolute risks of vascular dementia and all-cause dementia were significantly higher in individuals with high Lp(a) levels (*p* = 0.01 in both cases), although this was not the case for AD. Interestingly, the results did not reach statistical significance in the Copenhagen cohorts, with respective *p*-values of 0.42 and 0.48. However, they revealed a nuanced relationship involving the Lp(a) isoform size. In the Copenhagen studies, individuals with small LPA KIV-2 repeat numbers (≤5th percentile) compared to those with >50th percentile showed a sub-distribution hazard ratio for AD of 1.25 (95% CI 1.06–1.46) [36]. This finding suggests that, while the Lp(a) concentration per se may not influence the AD risk, the structural characteristics of the apo(a) component, which inversely correlate with Lp(a) levels, may play a role in disease pathogenesis. 

These observed differences could be explained by differences either in the cohort studied or in the methodology used. However, they may also be a consequence of the different effects of Lp(a) on the vascular beds. Indeed, the relationship between Lp(a) and cerebrovascular diseases demonstrates a striking vessel size-dependent dichotomy. Mendelian randomization studies have established that genetically elevated Lp(a) levels are positively associated with large-vessel stroke (OR: 1.20, 95% CI: 1.11–1.30) per one-standard-deviation increase in Lp(a) [33]. This association reflects Lp(a)’s pro-atherogenic properties in large arteries, where it promotes atherosclerotic plaque formation through multiple mechanisms, including lipid deposition, inflammation, and impaired fibrinolysis. A meta-analysis of 41 studies confirmed the significant association between elevated Lp(a) and large-artery atherosclerosis ischemic stroke, as well as intracerebral hemorrhage, which represent risk factors for AD [33]. Conversely, small-vessel stroke exhibits an inverse association with Lp(a) levels, (OR: 0.92, 95% CI: 0.88–0.97) per one-standard-deviation increase [33]. This paradoxical protective effect in small-vessel disease suggests distinct pathophysiological mechanisms operating in different vascular beds, with a protective role in the capillaries. However, the Reasons for Geographic and Racial Differences in Stroke (REGARDS) study identified elevated Lp(a) as a risk factor for ischemic stroke overall, with a notably stronger effect observed in Black individuals, reflecting both higher baseline Lp(a) levels and potentially differential genetic susceptibility in this population [37]. These vessel-specific effects highlight the complexity of Lp(a)’s vascular biology and raise important questions about its potential role in cerebral small-vessel disease and related neurodegenerative pathologies, which require further study. It would be particularly important to investigate AD in mice that express/overexpress human *LPA* as wild-type mice do not express apo(a) [38].

## 4. High-Density Lipoprotein and Alzheimer’s Disease: Cholesterol and Apolipoprotein Levels

HDL was first characterized as the main regulator of reverse cholesterol transport, the process by which excess cholesterol is removed from foam cells in atherosclerotic lesions and transported to the liver for excretion. Due to its role in reverse cholesterol transport, it was believed that increasing HDL levels would be beneficial in the context of cardiovascular disease [39]. However, evidence has shown that the relationship between HDL-C levels and cardiovascular disease is U-shaped, where both very low and very high levels of HDL-C are associated with an increased risk of cardiovascular mortality [6]. HDL biogenesis is initiated by the secretion and lipidation of apoA-I by hepatocytes and enterocytes. However, HDL particles that circulate in the blood are heterogeneous in size (6–17 nm), density (1.063 to 1.210 g/mL), and composition [10]. In addition to apoA-I and cholesterol, HDL particles harbor over 250 lipid and protein species [40,41]. These proteins and lipids are not uniformly present on all HDL particles, but, rather, different mixtures of proteins and lipids form heterogeneous HDL subpopulations with specific functions, including anti-thrombotic, anti-oxidant, anti-inflammatory, and cytoprotective properties [42]. These functions make HDL beneficial in a wide range of diseases, not just cardiovascular diseases [10], and epidemiological evidence suggests that circulating HDL may attenuate the risk of neurodegenerative diseases such as AD.

We and others have found that deleting apoA-I in AD mice drastically reduces the HDL levels and exacerbates CAA and cerebrovascular inflammation [43,44], although other groups have reported no effect [45] or the opposite result [46]. Conversely, genetic *APOA1* overexpression reduces CAA and neuroinflammation [47]. Further supporting HDL’s function against AD, the systemic delivery of lipid-free apoA-I Milano—an apoA-I mutant that also reduces the risk of ASCVD—acutely leads to long-lasting reductions in CAA and neuroinflammation [48]. Finally, we observed an acute reduction in Aβ deposition in the brain in the AD mouse model APP/PS1 after the injection of reconstituted HDL (rHDL), which is composed of human apoA-I and 1,2-dioleoyl-sn-glycerol-phosphocholine (DOPC) [49]. Taken together, these observations provide proof of concept that blood-borne HDL can affect AD-relevant outcomes, at least in preclinical models. 

These preclinical studies were supported by early epidemiological studies that demonstrated an inverse association between HDL and apoA-I levels and the risk of AD. The level of apoA-I, the major apolipoprotein found on HDL particles, was positively correlated with cognitive scores and high serum HDL-C (>1.42 mmol/L) in cognitively normal elderly individuals and was associated with a reduced AD risk (HR, 0.4), even after adjusting for the *APOE* genotype and vascular risk factors [50,51]. More recent analyses have found that, as with cardiovascular disease and all-cause mortality, HDL-C levels demonstrate a U-shaped relationship with AD risk [52,53]. A recent study of the Aspirin in Reducing Events in the Elderly (ASPREE) trial, which involved 18,668 initially healthy older adults, found that high plasma HDL-C levels (>4.4 mmol/L) were associated with a 27% higher risk of incident dementia (HR: 1.27, 95% CI: 1.03–1.58) [52]. This risk was particularly pronounced in participants aged 75 years and older (HR: 1.42, 95% CI: 1.10–1.83). The relationship between HDL-C and cognitive function exhibits striking sex differences [52]. A cross-sectional study using the National Health and Nutrition Examination Survey (NHANES) between 2011 and 2014 with 2777 participants aged ≥60 years revealed a significant nonlinear, inverted U-shaped association between serum HDL-C and cognitive function. Moderate levels were associated with better cognitive function, while low and high levels were linked to cognitive decline [54]. In men, HDL-C showed positive associations across all cognitive domains (e.g., delayed recall: β = 0.10, 95% CI: 0.04–0.17, *p* < 0.001). In women, however, these associations were weaker or insignificant, and an inverted U-shaped pattern predominated [54]. However, sensitivity analyses attenuated this finding.

Mendelian randomization studies have produced conflicting results, with some suggesting that HDL-C levels are not a cause of AD risk [55,56] and others indicating a protective association between genetically predicted HDL-C levels and AD risk (OR: 0.51, 95% CI: 0.29–0.89). However, it is important to note that these studies only address a causal link between disease risk and elevated HDL-C levels mediated by particular genes. They do not take into account the complex changes in HDL function and composition that can occur during disease and can be superior predictors of risk [10,54,57,58,59]. Recently, two large genome-wide association studies (GWAS) for AD identified several genes encoding components of HDL or mediating HDL metabolism or function as significantly associated with AD risk, e.g., *APOA1*, *APOA2*, *APOC1*, *APOE*, *APOM*, *PON1*, *CLU*, *LCAT*, *CETP*, and *ABCA1* [60,61]. Together, these observations suggest a potential link between HDL and AD that warrants further investigation. 

**Figure 2:**
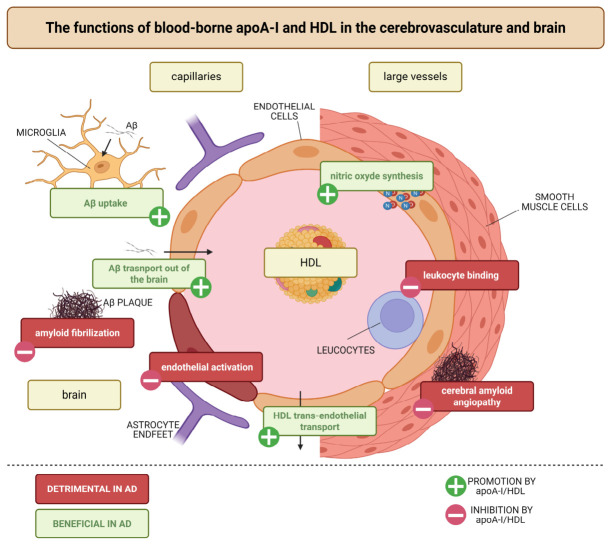
Blood-borne HDL and apolipoprotein A-I have several beneficial functions in the cerebrovasculature and brain. By interacting with the cerebrovasculature or entering the brain via trans-endothelial transport, HDL reduces several detrimental events in Alzheimer’s disease (AD; red) or promotes beneficial actions (green). Notably, HDL reduces endothelial activation and subsequent leukocyte binding and diapedesis. HDL promotes the transport of Aβ40 and Aβ42 through endothelial cells, reducing both parenchymal plaque formation and cerebral amyloid angiopathy (CAA). HDL delays Ab40 and Ab42 fibrillation. HDL and its principal apolipoprotein, apoA-I, enter the brain, where they stimulate the uptake of Aβ42 fibrils by microglia. Finally, HDL induces the release of nitric oxide (NO) by endothelial cells, thereby promoting vasorelaxation. Aβ = beta-amyloid, apoA-I = apolipoprotein A-I, HDL = high-density lipoprotein [49,77,78,85]. BioRender.com. License Robert J. (2026).

Notably, the paradoxical associations between HDL-C and cognitive outcomes may be partially explained by the heterogeneity in HDL particle composition and function [10]. HDL comprises multiple subfractions with distinct biological properties. Research using quantitative nuclear magnetic resonance (NMR)-based lipoprotein analysis identified elevated HDL-4 subfractions and triglycerides in the serum of patients with AD, with HDL-4 levels being particularly high in women with mild cognitive impairment [62]. HDL-4 parameters and various triglycerides correlated positively with AD pathology. In addition to particle size, HDL content may also play an important role, as previously thought. HDL containing apoE represents only ~9% of the total plasma HDL and was previously believed to mainly serve as a ligand for the hepatic uptake of HDL [62]. However, HDL containing apoE is now considered to promote critical steps in reverse cholesterol transport [63,64] and has been confirmed as a potential biomarker for ASCVD [65,66]. An epidemiological study recently showed that the level of HDL containing apoE particles and lacking apoC-III inversely correlates with the risk of AD [67]. Whether other HDL subpopulations might have a similar role remains to be investigated. In particular, HDL containing apoM might be of interest because, despite representing only a small percentage of the total blood-borne HDL, it modulates several anti-inflammatory processes of HDL via its lipid, sphingosine 1-phosphate (S1P) [68,69,70,71,72,73,74,75]. HDL function or dysfunction in disease may therefore be a better prognostic marker than HDL-C. Notably, the cholesterol efflux capacity and lecithin-cholesterol acyl transferase (LCAT) activity of HDL appear critical for neuroprotection. One study examined 194 participants and found that the HDL cholesterol efflux capacity and LCAT activity were reduced in *APOE4* carriers, regardless of diagnosis, and in *APOE3* carriers with AD [76]. LCAT activity and the particle size were positively correlated with neuropsychological scores and negatively correlated with clinical dementia ratings. The HDL particle size was significantly smaller in patients with AD and mild cognitive impairment compared to controls, particularly in *APOE4* carriers [76]. These findings suggest that it is HDL dysfunction, rather than low HDL-C levels alone, that contributes to neurodegeneration. In vitro experiments offer the possibility of defining these functions specifically by HDL subtype. Notably, we and others have identified at least four protective functions of HDL in AD (Figure 2). The first is the reduction of Aβ40- and Aβ40-induced inflammation of the endothelium. This function depends on the HDL receptor scavenger receptor BI (SR-BI) [77]. The second is the activation of endothelial nitric oxide synthase (eNOS) and the subsequent induction of nitric oxide (NO) production by brain endothelial cells [78]. Thirdly, HDL delays Aβ40 and Aβ42 fibrillization, a function that we later confirmed is conserved among different lipoprotein species, as it is also exerted by LDL [79]. This function may be due to the ability of lipoproteins to bind hydrophobic Aβ peptides [80,81]. Fourthly, it inhibits CAA by increasing Aβ40 and Aβ42 transport through the vasculature [43,78,79]. Interestingly, this protective function is independent of SR-BI but dependent on the presence of apoE in HDL and requires HDL to enter the cerebrovasculature. These results are counterintuitive, as SR-BI has been reported to limit HDL transport through aortic and brain endothelial cells in vitro [82,83]. However, and in contrast to these in vitro observations, the level of apoA-I, which originates exclusively from the blood, is not reduced in the brains of mice lacking SR-BI [84]. Together, these results suggest that HDL transport into the brain may be regulated by multiple receptors, and we recently showed that LDLR regulates HDL transport through brain endothelial cells in vitro and in vivo and that LDLR preferentially facilitates the transport of HDL containing apoE [82]. These results are particularly important because blood-borne apoA-I has been found to colocalize with microglia and activate their Aβ endocytosis activity [85]. Taken together, these results suggest that identifying the appropriate HDL subpopulation could facilitate the development of HDL-based AD therapeutics or HDL-based carriers to pass the BBB.

## 5. Lipid-Lowering Medications and Alzheimer’s Disease

Several drugs are currently on the market or in clinical trials to modulate the cholesterol levels in plasma. These include statins, PCSK9 inhibitors, ANGPTL inhibitors, and CETP and LP(a) inhibitors. In the next section, we will review the current evidence for the use of these drugs in AD and other neurodegenerative disorders.

### 5.1. Statins

Statins inhibit HMG-CoA reductase, thereby reducing endogenous cholesterol synthesis, and are among the most widely prescribed medications globally. Due to their significant impacts on lipid metabolism and potential pleiotropic properties, statins have undergone extensive research for their neuroprotective potential, with mixed outcomes. Early GWAS studies revealed that the rs3846662_G SNP, which increases full-length HMG-CoA reductase production by altering exon 13 splicing and raising LDL-C levels, is associated with an increased risk of AD and an earlier age of onset [86]. These results were later confirmed in two studies: one analyzing a Han Chinese cohort [87] and another one analyzing the Quebec Founder Population (QFP), the Alzheimer’s Disease Cooperative Study (ADCS), and the Alzheimer’s Disease Neuroimaging Initiative (ADNI) cohorts. In all studies, the rs3846662_A allele was found to be protective against AD, as well as in the conversion from mild cognitive impairment to AD [88]. Together, these studies support the idea that inhibiting HMG-CoA reductase via statins would be beneficial in the treatment of AD. However, drug-targeted mendelian randomization on *HMGCR* generated conflicting results. Analyses of the International Genomics of Alzheimer’s Project (IGAP) and the Psychiatric Genomics Consortium (PGC), which included a total of 24,718 AD patients and 56,685 control participants of European ancestry, failed to identify genetic support for repurposing statins [89]. Similarly, analyses of the CGPS and CCHS, which included 111,194 individuals from the Danish population, including 1001 participants with AD, found no benefit associated with a 1 mmol/L reduction in LDL-C for the risk of AD. However, they did find a reduced risk of vascular dementia [90]. A Mendelian randomization study further examined how the genetic inhibition of *HMGCR* affects cognitive function. Using data from 172,082 individuals of European ancestry from the ADNI, UKB, Global Lipid Genetics Consortium (GLGC), Enhancing Neuroimaging Genetics through Meta-Analysis (ENIGMA), and Coronary Artery Disease Genome-Wide Replication and Meta-Analysis (CARDIoGRAM) studies, plus the Coronary Artery Disease (C4D) Genetics (CARDIoGRAMplusC4D) study, the authors found that the genetic inhibition of HMGCR showed a harmful impact on cognitive performance scores (β = −0.082, 95% CI: −0.16 to −0.0079), reaction time (β = 0.00064, 95% CI: 000030 to 0.00098), and the cortical surface area (β = −0.18; 95% CI: −0.35 to −0.014) [11]. Interestingly, while genetically lowering LDL-C via *HMGCR* did not reduce the risk of AD, a recent study of 1,091,775 individuals from the UKB, CGPS, und CCHS found that the genetic lowering of non-HDL cholesterol reduces the risks of all types of dementia, vascular dementia, unspecified dementia, and AD [91]. The inclusion of the FinnGen study and the GLGC in a two-sample Mendelian randomization study confirmed these results for all types of dementia but not for other types of dementia, including AD. Multiple large-scale observational studies and meta-analyses have suggested protective associations between statin use and dementia risk. A systematic review and meta-analysis of 57 observational studies found that statin use was associated with a 20% decreased risk of dementia (OR: 0.80; 95% CI: 0.75–0.86) and a 32% decreased risk of AD (OR: 0.68; 95% CI: 0.56–0.81) [92]. Similar risk reductions were observed with both lipophilic and hydrophilic statins, while high-potency statins demonstrated greater efficacy, with a 20% reduction, compared to a 16% reduction with low-potency statins. Another comprehensive review reported that statin use was associated with a 17% lower risk of all-cause dementia and a 31% lower risk of AD [93]. Despite these encouraging observational findings, randomized controlled trials have produced more ambiguous results. A systematic review and meta-analysis of 20 randomized controlled trials involving 139,169 participants found that lipid-lowering therapy, predominantly statins, was not significantly associated with a reduction in dementia or cognitive impairment (OR: 0.96; 95% CI: 0.74–1.26) over a mean follow-up period of 34.5 months [94]. Subgroup analyses showed no significant association with statins (OR 0.90, 95% CI 0.67–1.21). The discrepancy between genetic studies, observational studies, and randomized controlled trials may reflect one or more of the following: (1) healthy user bias in observational studies, (2) insufficient follow-up durations in randomized controlled trials, (3) the timing of the intervention (midlife treatment may be more effective), and (4) heterogeneous treatment effects.

Despite the conflicting results for AD, statins might also be effective against other neurodegenerative diseases. Notably, a study using consolidated data from three observational cohorts found that statin use was protective against both cerebral atherosclerosis and incident parkinsonism in older adults [95]. Atherosclerosis accounted for 17% of the risk of developing Parkinson’s disease, and statins were found to reduce the risk of developing the condition rather than slowing the progression of symptoms. A four-year retrospective observational cohort study of 104 patients with de novo Parkinson’s disease found that long-term statin users showed lower motor deterioration compared to nonusers, with slower progression of rigidity scores [96]. However, a prospective cohort study with multi-omics analyses found that statins may accelerate cognitive decline in patients with Parkinson’s disease [97].

### 5.2. Proprotein Convertase Subtilisin/Kexin Type 9 Inhibitors

PCSK9 inhibitors are a newer class of lipid-lowering agents that dramatically reduce LDL-C levels by preventing the degradation of LDLR in hepatocytes, thereby increasing LDL turnover. While the role of PCSK9 in cardiovascular disease is well established, its role in neurodegenerative diseases is still being debated. Animal studies consistently demonstrate that elevated PCSK9 levels promote the accumulation of Aβ plaques, neuroinflammation, and cognitive decline, while the genetic deletion or pharmacological inhibition of PCSK9 mitigates these effects [98,99]. However, clinical studies have produced conflicting results. An early GWAS study investigated the rs11591147 SNP, which was associated with 10–16% lower LDL-C levels in the Prospective study of Pravastatin in the Elderly at Risk (PROSPER) study of 5777 participants from Scotland, Ireland, and the Netherlands. This study found no association with cognitive performance [100]. A Mendelian randomization study of 111,194 participants from the CGPS and CCHS found no association between low levels of LDL-C due to PCSK9 and the risk of AD, vascular dementia, or any type of dementia [90]. Similarly, and in contrast to what was observed with *HMCGR*, a Mendelian randomization study of 1,091,775 individuals from the UKB, CGPS, and CCHS found that genetically lowering non-HDL-C did not affect the risks of AD, all types of dementia, and vascular dementia but reduced that of unspecified dementia [91]. Finally, in another Mendelian randomization study with 172,082 individuals of European ancestry, the authors found no genetic evidence of PCSK9 inhibition in cognitive function (e.g., cognitive performance, memory performance, reaction time, deficit fluid intelligence score, average cortical thickness, and total cortical surface area) or dementia (e.g., disease progression score, hippocampus volume, cerebral amyloid deposition, and Lewy body dementia) [11], suggesting no benefit, but also no harm, in targeting PCSK9 in the context of AD. On the other hand, a Mendelian randomization study of the IGAP and PGC cohorts, which included 24,718 AD patients and 56,685 controls from Europe and North America, found that PCSK9 inhibition increased the risk of AD (OR: 1.45, 95% CI: 0.123–1.69) across both studies [89]. In the Framingham Heart Study Offspring cohort (Gen 2), which included 3487 participants with genetic data on *PCSK9* and *APOE*, it was revealed that a higher concentration of plasma PCSK9 was associated with a lower risk of AD (HR: 0.74; 95% CI: 0.58, 0.94) in non-*APOE4* carriers. There was no association in *APOE4* individuals [101]. These findings were further validated in the ADNI, which included 1476 participants and showed that *PCSK9* genotypes were associated with AD risk and the AD biomarker, a low Aβ42 concentration in CSF, only in APOE ε4 non-carriers. The role of PCSK9 has also been investigated in other neurodegenerative diseases. One study found that low PCSK9 levels marginally reduced the risk of amyotrophic lateral sclerosis (ALS) (OR: 0.89; 95% CI: 0.77–1.00) but increased the risk of Parkinson’s disease (OR: 1.417; 95% CI: 1.178–1.657) [102]. In this study, the genetic proxies for *HMGCR* inhibition (statin targets) increased the risk of Parkinson’s disease (OR: 1.907; 95% CI: 1.502–2.312). The discrepancy between these studies may be due to the sizes of the cohorts studied, but also their ethnicities and the number of SNPs studied [98]. Nonetheless, these observations suggest that long-term PCSK9 inhibition may have an adverse effect on cognition. While these effects may be outweighed by the cardiovascular benefits, they nonetheless warrant pharmacovigilance. In this regard, it is important to note that no neurodegenerative events were reported in a clinical trial of the PCSK9 inhibitor evolocumab, which included 27,564 patients over a median follow-up period of 2.2 years [103]. These observations were confirmed in a second cohort of 158 Spanish patients who were followed for two years with the addition of a second inhibitor, alirocumab [104]. Together, these studies suggest that PCSK9 inhibition does not affect cognition in the short term; however, longer follow-up studies are needed to draw solid conclusions.

### 5.3. Cholesteryl Ester Transfer Protein Inhibitors

CETP facilitates the transfer of cholesteryl esters from HDL to apoB-containing lipoproteins. CETP inhibition raises HDL-C and lowers LDL-C, making CETP an attractive therapeutic target. Several CETP inhibitors have been developed, but most were discontinued when they showed no improvement in cardiovascular outcomes despite raising HDL-C levels [105]. However, the role of CETP has recently become a focus of interest in AD, as preclinical studies and human genetic evidence suggest that modulating CETP levels may provide neuroprotective benefits [106]. Notably, a drug-target Mendelian randomization analysis of neurodegenerative diseases found that lower CETP activity was associated with a significantly reduced risk of Lewy body dementia (OR: 0.81; 95% CI: 0.74–0.89) and Parkinson’s dementia (OR: 0.26; 95% CI: 0.14–0.48) [107]. This protective effect was more pronounced in *APOE4* carriers for Lewy body dementia (OR: 0.61; 95% CI: 0.51–0.73). Similarly, a Mendelian randomization study of the UKB, CGPS, and CCHS found a reduced risk of AD, vascular dementia, unspecified dementia, and all types of dementia with a 1 mmol/L reduction in LDL-C. These associations remained in a two-sample Mendelian randomization study after the addition of the FinnGen and GLGC cohorts [91]. Lower CETP activity was also associated with a higher total brain volume (0.04 per standard deviation, 95% CI: 0.02–0.06). Experimental validation comes from a study using CETP transgenic mice crossed with a mouse model of amyloidosis. Administering the CETP inhibitor evacetrapib in these mice maintained memory, independently of classic AD markers [108]. This study establishes CETP as a novel AD treatment target and proposes repurposing CETP inhibitors to delay or prevent cognitive impairment. The independence from classic AD markers suggests that CETP inhibition may operate through alternative mechanisms, perhaps on the cerebrovasculature. Recently, phase 2 and phase 3 clinical trials (BROADWAY) were conducted on the CETP inhibitor Obicetrapib for AD. These trials showed that a daily dose of 10 mg of Obicetrapib significantly slowed the progression of AD biomarkers, such as p-tau217, over 12 months, with the most pronounced effects observed in *APOE4* carriers [109]. Interestingly, these results were not associated with a rise in HDL-C but to a decrease in LDL-C. Together, these results suggest that CETP inhibition may be a viable strategy for preventing AD. I recommend reading the review by Poliakova and Wellington for further information [106].

### 5.4. Angiopoietin-like Proteins

ANGPTL3 and ANGPTL4 are hepatic secreted proteins that regulate lipid metabolism by inhibiting lipoprotein lipase and endothelial lipase. ANGPTL inhibitors are in clinical development for the treatment of severe hypertriglyceridemia and familial hypercholesterolemia [110,111]. Although these proteins have been shown to effectively reduce total, LDL, and non-HDL-C levels, their role in AD or other dementia remains largely unexplored. The largest Mendelian randomization study so far investigated 1,091,775 individuals from the UKB, CGPS, and CCHS and found that genetically lowering non-HDL-C via ANGPTL4 did not affect the risks of AD, all types of dementia, vascular dementia, and unspecified dementia [91]. The role of ANGPTL3 was unfortunately not investigated in this study. The role of ANGPTL3 was investigated in another Mendelian randomization study examining the relationship between lipid metabolism and Lewy body dementia, which found that a low level of ANGPTL3 was significantly associated with an increased risk [112]. Another study investigated the therapeutic potential of recombinant ANGPTL4 in Parkinson’s disease using in vivo and in vitro experimental models [113]. This study demonstrated mechanistic effects and biomarker changes in the presence of ANGPTL4, suggesting therapeutic potential in Parkinson’s disease. This is interesting, as ANGPTL4 administration may have direct neuroprotective effects independently of its effects on lipid metabolism. As these agents advance in the clinic, careful monitoring of neurological outcomes will be essential.

### 5.5. Lipoprotein (a) Inhibitors

The development of potent Lp(a)-lowering therapies has advanced rapidly, driven by the established causal role of Lp(a) in cardiovascular disease. Multiple therapeutic modalities are now in late-stage clinical development, achieving unprecedented degrees of Lp(a) reduction exceeding 80% [9]. However, the neurological implications of pharmacological Lp(a)-lowering remain entirely unexplored. Because of the uncertainties in the effects of Lp(a), with the potential beneficial effects of higher plasma levels, careful monitoring of neurological outcomes will be essential during clinical trials. Importantly, despite the conflicting results regarding high plasma levels of Lp(a), Thomas and colleagues report no detrimental effects with low levels, suggesting a reduced risk for patients under these drugs [36]. 

Other emerging therapies include apoC-III inhibitors, which are being developed to treat severe hypertriglyceridemia [114]. Due to the association between apoC-III in non-HDL fractions and AD pathology [115], inhibiting apoC-III represents a potential therapeutic avenue. However, the neurological effects have not yet been systematically studied. Finally, HDL-based therapies have been tested in the context of AD and other neurodegenerative diseases. Several formulations with different proteins have been tested, including apoA-I, apoE, and clusterin (apoJ) [39]. All have demonstrated positive outcomes in preclinical AD models, particularly in mice overexpressing APP/PS1 genes. Whether similar outcomes will be seen in other AD models or in patients remains to be seen. We recommend reading the cited reviews for further insights into HDL therapeutics [7,39].

## 6. Discussion

The complex relationships between peripheral lipoproteins and brain health likely reflect multiple interconnected mechanisms. While most studies suggest that lowering non-HDL-C levels reduces the risk of AD, the exact mechanisms remain poorly understood, and drugs targeting non-HDL-C have shown mixed results. Further research is needed to determine whether the effect is direct, particularly by acting on the cerebrovasculature, or whether it acts by reducing the risk of ASCVD. Lipid-lowering medications may influence neurodegeneration through lipid-dependent and pleiotropic mechanisms. Notably, the anti-inflammatory, antioxidant, and vascular-protective effects of statins may contribute to neuroprotection beyond lowering LDL-C [116]. However, excessive cholesterol depletion could impair synaptic function [4]. On the other hand, HDL and apoA-I exert neuroprotective effects through anti-inflammatory, antioxidant, and Aβ-clearing mechanisms. However, HDL dysfunction can transform HDL from protective to pro-inflammatory particles [57,58,59,117]. The paradoxical associations between high HDL-C levels and an increased risk of dementia may reflect HDL dysfunction rather than quality. In this regard, rHDL therapies could be particularly interesting if we produce particles with the optimal functional composition.

Observations from epidemiological studies have important clinical implications. First, due to the long latency period between exposure to vascular risk factors and the onset of dementia, managing lipids in midlife may be more important for preventing AD than interventions in late life. Second, aggressively lowering LDL-C in midlife may provide dual benefits for cardiovascular and brain health. Third, the nonlinear relationships between lipids and cognitive outcomes suggest that the optimal lipid targets for neuroprotection may differ from those for cardiovascular protection. Fourth, apolipoprotein measurements (apoA-I, apoB, apoE, and the apoB/apoA-I ratio) may provide better dementia risk stratification than traditional lipid panels (total-C, LDL-C, HDL-C, non-HDL-C). The strong association between apoB/apoA-I ratios and dementia risk suggests that this metric could be incorporated into dementia risk assessment tools [22]. Similarly, developing a clinical method to measure HDL function instead of measuring HDL-C would be ultimately more informative. Fifth, *APOE* genotyping, being the strongest genetic risk factor for late-onset AD, may help to identify individuals who would benefit most from lipid-modulating interventions. For example, the stronger protective effects of CETP inhibition in *APOE4* carriers suggest the potential for precision medicine approaches [109]. Sixth, evidence regarding the effects of drugs lowering LDL-C and non-HDL-C on dementia is mixed, suggesting that the benefits may be concentrated in specific subgroups (genetic, ethnicity, or sex) or require longer treatment durations. 

There are several important limitations that warrant discussion. First, while vascular mechanisms are well established, the extent to which peripheral lipids directly influence brain cholesterol metabolism is debated in particular, as it is believed that cholesterol is not transported through the BBB [118]. However, we and others have found that blood-borne HDL is transported through the BBB and found within the brain vasculature and parenchyma [82,83,119]. Whether HDL particles cross as intact particles in vivo remains to be investigated, and this will shed new light on the potential lipid exchange between the blood and the brain. Second, the potential adverse effects of very low cholesterol levels on brain function require careful consideration. Although aggressive LDL-C lowering appears to be safe in cardiovascular trials, the long-term neurological effects of maintaining very low LDL-C remain unknown. Finally, substantial but poorly understood differences between the sexes exist regarding the relationship between lipids and cognition. The stronger associations between high HDL-C and cognitive impairment in females suggest that lipid management strategies may require sex-specific tailoring. Similar considerations should be given to ethnicity.

These observations reveal several key research priorities, including the need for long-term, randomized, controlled trials of lipid-lowering therapies. Large-scale studies with over a decade of follow-up, beginning in middle age, are necessary to determine if these medications can prevent AD. Novel treatments, including CETP, ANGPTL, Lp(a), and apoC-III inhibitors, warrant further investigation in dementia prevention trials due to promising genetic and experimental evidence. Additionally, long-term safety surveillance is critical for monitoring neurological outcomes in patients receiving emerging lipid-lowering therapies, and precision medicine approaches are important as well. Research should focus on identifying the subgroups that are most likely to benefit from lipid-modulating interventions based on factors such as age, sex, *APOE* genotype, baseline lipid levels, and other risk indicators. Mechanistic studies are needed to clarify how peripheral lipids affect brain pathology in order to develop targeted therapies. However, a mouse model may not always be the best way to answer these questions, because mice and humans have different plasma lipid profiles. While humans transport cholesterol primarily via LDL, mice rely on HDL and lack CETP and Lp(a) [39,106].

In conclusion, the relationship between lipoproteins and brain health is complex, multifaceted, and context-dependent. Elevated levels of LDL-C and apoB, particularly in midlife, are associated with an increased risk of dementia and a greater burden of AD neuropathology. This supports the idea that LDL-C and nonHDL-C are modifiable risk factors. However, HDL-C exhibits paradoxical associations with cognitive outcomes, as both very low and very high levels are potentially detrimental. HDL function and HDL-subpopulation, rather than HDL-C concentration alone, appear critical for neuroprotection. However, genetic, observational, and clinical studies often produce conflicting results regarding lipid-lowering medications. These discrepancies will need to be investigated further in follow-up studies, particularly regarding the optimal timing and duration of lipid-lowering interventions, the mechanisms linking peripheral lipids to brain pathology, and the identification of individuals most likely to benefit (based on age, sex, ethnicity, and *APOE* genotype). Large-scale, and long-term trials initiated in midlife, combined with mechanistic studies and precision medicine approaches, are needed to realize the full potential of lipid modulation for AD prevention. Nevertheless, understanding and targeting lipid metabolism represents a promising approach to the prevention and treatment of AD, particularly since several medications for treating ASCVD are already on the market or in development.

## Data Availability

No new data were created or analyzed in this study. Data sharing is not applicable to this article.

## References

[1] World Health Organization (2021). Global Status Report on the Public Health Response to Dementia.

[2] Martin M.G., Pfrieger F., Dotti C.G. (2014). Cholesterol in brain disease: Sometimes determinant and frequently implicated. EMBO Rep..

[3] Jin U., Park S.J., Park S.M. (2019). Cholesterol Metabolism in the Brain and Its Association with Parkinson’s Disease. Exp. Neurobiol..

[4] Shin K.C., Ali Moussa H.Y., Park Y. (2024). Cholesterol imbalance and neurotransmission defects in neurodegeneration. Exp. Mol. Med..

[5] Mortensen M.B., Dzaye O., Botker H.E., Jensen J.M., Maeng M., Bentzon J.F., Kanstrup H., Sorensen H.T., Leipsic J., Blankstein R. (2023). Low-Density Lipoprotein Cholesterol Is Predominantly Associated with Atherosclerotic Cardiovascular Disease Events in Patients with Evidence of Coronary Atherosclerosis: The Western Denmark Heart Registry. Circulation.

[6] von Eckardstein A., Nordestgaard B.G., Remaley A.T., Catapano A.L. (2023). High-density lipoprotein revisited: Biological functions and clinical relevance. Eur. Heart J..

[7] Button E.B., Robert J., Caffrey T.M., Fan J., Zhao W., Wellington C.L. (2019). HDL from an Alzheimer’s disease perspective. Curr. Opin. Lipidol..

[8] Zhang H., Temel R.E., Martel C. (2014). Cholesterol and lipoprotein metabolism: Early Career Committee contribution. Arter. Thromb. Vasc. Biol..

[9] Greco A., Finocchiaro S., Spagnolo M., Faro D.C., Mauro M.S., Raffo C., Sangiorgio G., Imbesi A., Laudani C., Mazzone P.M. (2025). Lipoprotein(a) as aPharmacological Target: Premises, Promises, and Prospects. Circulation.

[10] von Eckardstein A., Robert J. (2026). HDL Are HostDefense Lipoproteins. Circ. Res..

[11] Rosoff D.B., Bell A.S., Jung J., Wagner J., Mavromatis L.A., Lohoff F.W. (2022). Mendelian Randomization Study of PCSK9 and HMG-CoA Reductase Inhibition and Cognitive Function. J. Am. Coll. Cardiol..

[12] Tan J.S., Hu M.J., Yang Y.M., Yang Y.J. (2021). Genetic Predisposition to Low-Density Lipoprotein Cholesterol May Increase Risks of Both Individual and Familial Alzheimer’s Disease. Front. Med..

[13] Wu Y., Chen F., Zhang T., Miao M., Zhang M., Zhang J., Chang E. (2025). The causal association between circulating metabolites and Alzheimer’s disease: A systematic review and meta-analysis of Mendelian randomization studies. Metabolomics.

[14] Power M.C., Rawlings A., Sharrett A.R., Bandeen-Roche K., Coresh J., Ballantyne C.M., Pokharel Y., Michos E.D., Penman A., Alonso A. (2018). Association of midlife lipids with 20-year cognitive change: A cohort study. Alzheimer’s Dement..

[15] Schilling S., Tzourio C., Soumare A., Kaffashian S., Dartigues J.F., Ancelin M.L., Samieri C., Dufouil C., Debette S. (2017). Differential associations of plasma lipids with incident dementia and dementia subtypes in the 3C Study: A longitudinal, population-based prospective cohort study. PLoS Med..

[16] Reitz C., Luchsinger J., Tang M.X., Manly J., Mayeux R. (2005). Impact of plasma lipids and time on memory performance in healthy elderly without dementia. Neurology.

[17] Reitz C., Tang M.X., Schupf N., Manly J.J., Mayeux R., Luchsinger J.A. (2010). Association of higher levels of high-density lipoprotein cholesterol in elderly individuals and lower risk of late-onset Alzheimer disease. Arch. Neurol..

[18] Lee M., Lee K.J., Kim J., Lee D.Y., Park R.W., Rhee S.Y., Cha J.M., Yang H.J., Jang J.W., Jung S. (2025). Low-density lipoprotein cholesterol levels and risk of incident dementia: A distributed network analysis using common data models. J. Neurol. Neurosurg. Psychiatry.

[19] Lee Y.B., Kim M.Y., Han K., Kim B., Park J., Kim G., Hur K.Y., Kim J.H., Jin S.M. (2022). Association between cholesterol levels and dementia risk according to the presence of diabetes and statin use: A nationwide cohort study. Sci. Rep..

[20] Iwagami M., Qizilbash N., Gregson J., Douglas I., Johnson M., Pearce N., Evans S., Pocock S. (2021). Blood cholesterol and risk of dementia in more than 1.8 million people over two decades: A retrospective cohort study. Lancet Healthy Longev..

[21] Peters R., Xu Y., Antikainen R., Beckett N., Gussekloo J., Jagger C., Jukema J.W., Keinanen-Kiukaanniemi S., Ryden L., Skoog I. (2021). Evaluation of High Cholesterol and Risk of Dementia and Cognitive Decline in Older Adults Using Individual Patient Meta-Analysis. Dement. Geriatr. Cogn. Disord..

[22] Gong J., Harris K., Peters S.A.E., Woodward M. (2022). Serum lipid traits and the risk of dementia: A cohort study of 254,575 women and 214,891 men in the UK Biobank. eClinicalMedicine.

[23] Chou P.S., Chen S.C., Hsu C.Y., Liou L.M., Juan C.H., Lai C.L. (2023). The Association between Electronegative Low-Density Lipoprotein Cholesterol L5 and Cognitive Functions in Patients with Mild Cognitive Impairment. J. Pers. Med..

[24] Wingo A.P., Vattathil S.M., Liu J., Fan W., Cutler D.J., Levey A.I., Schneider J.A., Bennett D.A., Wingo T.S. (2022). LDL cholesterol is associated with higher AD neuropathology burden independent of APOE. J. Neurol. Neurosurg. Psychiatry.

[25] Kakava S., Schlumpf E., Panteloglou G., Tellenbach F., von Eckardstein A., Robert J. (2022). Brain Endothelial Cells in Contrary to the Aortic Do Not Transport but Degrade Low-Density Lipoproteins via Both LDLR and ALK1. Cells.

[26] Picard C., Nilsson N., Labonte A., Auld D., Rosa-Neto P., Ashton N.J., Zetterberg H., Blennow K., Breitner J.C.B., the Alzheimer’s Disease Neuroimaging Initiative (2022). Apolipoprotein B is a novel marker for early tau pathology in Alzheimer’s disease. Alzheimer’s Dement..

[27] Nordestgaard L.T., Christoffersen M., Afzal S., Nordestgaard B.G., Tybjaerg-Hansen A., Frikke-Schmidt R. (2021). Triglycerides as a Shared Risk Factor between Dementia and Atherosclerotic Cardiovascular Disease: A Study of 125 727 Individuals. Clin. Chem..

[28] Rosenson R.S., Cushman M., McKinley E.C., Muntner P., Wang Z., Vaisar T., Heinecke J., Tangney C., Judd S., Colantonio L.D. (2023). Association Between Triglycerides and Incident Cognitive Impairment in Black and White Adults in the Reasons for Geographic and Racial Differences in Stroke Study. J. Am. Heart Assoc..

[29] Nagga K., Gustavsson A.M., Stomrud E., Lindqvist D., van Westen D., Blennow K., Zetterberg H., Melander O., Hansson O. (2018). Increased midlife triglycerides predict brain beta-amyloid and tau pathology 20 years later. Neurology.

[30] Zhou Z., Ryan J., Tonkin A.M., Zoungas S., Lacaze P., Wolfe R., Orchard S.G., Murray A.M., McNeil J.J., Yu C. (2023). Association Between Triglycerides and Risk of Dementia in Community-Dwelling Older Adults: A Prospective Cohort Study. Neurology.

[31] Loffler T., Flunkert S., Havas D., Santha M., Hutter-Paier B., Steyrer E., Windisch M. (2013). Impact of ApoB-100 expression on cognition and brain pathology in wild-type and hAPPsl mice. Neurobiol. Aging.

[32] Nordestgaard B.G., Langsted A. (2024). Lipoprotein(a) and cardiovascular disease. Lancet.

[33] Pan Y., Li H., Wang Y., Meng X., Wang Y. (2019). Causal Effect of Lp(a) [Lipoprotein(a)] Level on Ischemic Stroke and Alzheimer Disease: A Mendelian Randomization Study. Stroke.

[34] Kunutsor S.K., Khan H., Nyyssonen K., Laukkanen J.A. (2016). Is lipoprotein (a) protective of dementia?. Eur. J. Epidemiol..

[35] Larsson S.C., Gill D., Mason A.M., Jiang T., Back M., Butterworth A.S., Burgess S. (2020). Lipoprotein(a) in Alzheimer, Atherosclerotic, Cerebrovascular, Thrombotic, and Valvular Disease: Mendelian Randomization Investigation. Circulation.

[36] Thomas P.E., Vedel-Krogh S., Nielsen S.F., Nordestgaard B.G., Frikke-Schmidt R., Kamstrup P.R. (2025). Lipoprotein(a) and risk of dementia: Findings from three cohort studies. Eur. Heart J..

[37] Arora P., Kalra R., Callas P.W., Alexander K.S., Zakai N.A., Wadley V., Arora G., Kissela B.M., Judd S.E., Cushman M. (2019). Lipoprotein(a) and Risk of Ischemic Stroke in the REGARDS Study. Arter. Thromb. Vasc. Biol..

[38] Assini J.M., Clark J.R., Youssef A., Xing C., Doerfler A.M., Park S.H., Saxena L., Yaseen A.B., Boren J., Gros R. (2023). High levels of lipoprotein(a) in transgenic mice exacerbate atherosclerosis and promote vulnerable plaque features in a sex-specific manner. Atherosclerosis.

[39] von Eckardstein A., Robert J. (2025). Endocytosis, Transcytosis, and Retroendocytosis of HDL: Mechanisms, Pathophysiology, and Options for Clinical Exploitation. Arter. Thromb. Vasc. Biol..

[40] Goetze S., Frey K., Rohrer L., Radosavljevic S., Krützfeldt J., Landmesser U., Bueter M., Pedrioli P.G.A., von Eckardstein A., Wollscheid B. (2021). Reproducible Determination of High-Density Lipoprotein Proteotypes. J. Proteome Res..

[41] Davidson W.S., Shah A.S., Sexmith H., Gordon S.M. (2022). The HDL Proteome Watch: Compilation of studies leads to new insights on HDL function. Biochim. Biophys. Acta (BBA)-Mol. Cell Biol. Lipids.

[42] Robert J., Osto E., von Eckardstein A. (2021). The Endothelium Is Both a Target and a Barrier of HDL’s Protective Functions. Cells.

[43] Button E.B., Boyce G.K., Wilkinson A., Stukas S., Hayat A., Fan J., Wadsworth B.J., Robert J., Martens K.M., Wellington C.L. (2019). ApoA-I deficiency increases cortical amyloid deposition, cerebral amyloid angiopathy, cortical and hippocampal astrogliosis, and amyloid-associated astrocyte reactivity in APP/PS1 mice. Alzheimer’s Res. Ther..

[44] Lefterov I., Fitz N.F., Cronican A.A., Fogg A., Lefterov P., Kodali R., Wetzel R., Koldamova R. (2010). Apolipoprotein A-I deficiency increases cerebral amyloid angiopathy and cognitive deficits in APP/PS1DeltaE9 mice. J. Biol. Chem..

[45] Fagan A.M., Christopher E., Taylor J.W., Parsadanian M., Spinner M., Watson M., Fryer J.D., Wahrle S., Bales K.R., Paul S.M. (2004). ApoAI deficiency results in marked reductions in plasma cholesterol but no alterations in amyloid-beta pathology in a mouse model of Alzheimer’s disease-like cerebral amyloidosis. Am. J. Pathol..

[46] Contu L., Carare R.O., Hawkes C.A. (2019). Knockout of apolipoprotein A-I decreases parenchymal and vascular beta-amyloid pathology in the Tg2576 mouse model of Alzheimer’s disease. Neuropathol. Appl. Neurobiol..

[47] Lewis T.L., Cao D., Lu H., Mans R.A., Su Y.R., Jungbauer L., Linton M.F., Fazio S., LaDu M.J., Li L. (2010). Overexpression of human apolipoprotein A-I preserves cognitive function and attenuates neuroinflammation and cerebral amyloid angiopathy in a mouse model of Alzheimer disease. J. Biol. Chem..

[48] Fernandez-de Retana S., Montanola A., Marazuela P., De La Cuesta M., Batlle A., Fatar M., Grudzenski S., Montaner J., Hernandez-Guillamon M. (2017). Intravenous treatment with human recombinant ApoA-I Milano reduces beta amyloid cerebral deposition in the APP23-transgenic mouse model of Alzheimer’s disease. Neurobiol. Aging.

[49] Robert J., Stukas S., Button E., Cheng W.H., Lee M., Fan J., Wilkinson A., Kulic I., Wright S.D., Wellington C.L. (2016). Reconstituted high-density lipoproteins acutely reduce soluble brain Abeta levels in symptomatic APP/PS1 mice. Biochim. Biophys. Acta.

[50] Song F., Poljak A., Crawford J., Kochan N.A., Wen W., Cameron B., Lux O., Brodaty H., Mather K., Smythe G.A. (2012). Plasma apolipoprotein levels are associated with cognitive status and decline in a community cohort of older individuals. PLoS ONE.

[51] Stukas S., Robert J., Wellington C.L. (2014). High-density lipoproteins and cerebrovascular integrity in Alzheimer’s disease. Cell Metab..

[52] Hussain S.M., Robb C., Tonkin A.M., Lacaze P., Chong T.T., Beilin L.J., Yu C., Watts G.F., Ryan J., Ernst M.E. (2024). Association of plasma high-density lipoprotein cholesterol level with risk of incident dementia: A cohort study of healthy older adults. Lancet Reg. Health West. Pac..

[53] Kjeldsen E.W., Thomassen J.Q., Juul Rasmussen I., Nordestgaard B.G., Tybjaerg-Hansen A., Frikke-Schmidt R. (2022). Plasma high-density lipoprotein cholesterol and risk of dementia: Observational and genetic studies. Cardiovasc. Res..

[54] Fan L., Jiang H., Zhang Z. (2025). Investigating causal effects of HDL-C on cognitive function through cross-sectional and Mendelian randomization analyses: Concentration-response patterns and clues for Alzheimer’s disease prevention. BioData Min..

[55] Ostergaard S.D., Mukherjee S., Sharp S.J., Proitsi P., Lotta L.A., Day F., Perry J.R., Boehme K.L., Walter S., Kauwe J.S. (2015). Associations between Potentially Modifiable Risk Factors and Alzheimer Disease: A Mendelian Randomization Study. PLoS Med..

[56] Proitsi P., Lupton M.K., Velayudhan L., Newhouse S., Fogh I., Tsolaki M., Daniilidou M., Pritchard M., Kloszewska I., Soininen H. (2014). Genetic predisposition to increased blood cholesterol and triglyceride lipid levels and risk of Alzheimer disease: A Mendelian randomization analysis. PLoS Med..

[57] Riwanto M., Rohrer L., Roschitzki B., Besler C., Mocharla P., Mueller M., Perisa D., Heinrich K., Altwegg L., von Eckardstein A. (2013). Altered activation of endothelial anti- and proapoptotic pathways by high-density lipoprotein from patients with coronary artery disease: Role of high-density lipoprotein-proteome remodeling. Circulation.

[58] Besler C., Heinrich K., Rohrer L., Doerries C., Riwanto M., Shih D.M., Chroni A., Yonekawa K., Stein S., Schaefer N. (2011). Mechanisms underlying adverse effects of HDL on eNOS-activating pathways in patients with coronary artery disease. J. Clin. Investig..

[59] Zewinger S., Kleber M.E., Rohrer L., Lehmann M., Triem S., Jennings R.T., Petrakis I., Dressel A., Lepper P.M., Scharnagl H. (2017). Symmetric dimethylarginine, high-density lipoproteins and cardiovascular disease. Eur. Heart J..

[60] Kunkle B.W., Grenier-Boley B., Sims R., Bis J.C., Damotte V., Naj A.C., Boland A., Vronskaya M., van der Lee S.J., Amlie-Wolf A. (2019). Genetic meta-analysis of diagnosed Alzheimer’s disease identifies new risk loci and implicates Abeta, tau, immunity and lipid processing. Nat. Genet..

[61] Jansen I.E., Savage J.E., Watanabe K., Bryois J., Williams D.M., Steinberg S., Sealock J., Karlsson I.K., Hagg S., Athanasiu L. (2019). Genome-wide meta-analysis identifies new loci and functional pathways influencing Alzheimer’s disease risk. Nat. Genet..

[62] Berezhnoy G., Laske C., Trautwein C. (2022). Quantitative NMR-Based Lipoprotein Analysis Identifies Elevated HDL-4 and Triglycerides in the Serum of Alzheimer’s Disease Patients. Int. J. Mol. Sci..

[63] Morton A.M., Furtado J.D., Mendivil C.O., Sacks F.M. (2019). Dietary unsaturated fat increases HDL metabolic pathways involving apoE favorable to reverse cholesterol transport. JCI Insight.

[64] Morton A.M., Koch M., Mendivil C.O., Furtado J.D., Tjønneland A., Overvad K., Wang L., Jensen M.K., Sacks F.M. (2018). Apolipoproteins E and CIII interact to regulate HDL metabolism and coronary heart disease risk. JCI Insight.

[65] Qi Y., Liu J., Wang W., Wang M., Zhao F., Sun J., Liu J., Zhao D. (2018). Apolipoprotein E-containing high-density lipoprotein (HDL) modifies the impact of cholesterol-overloaded HDL on incident coronary heart disease risk: A community-based cohort study. J. Clin. Lipidol..

[66] Sacks F.M., Liang L., Furtado J.D., Cai T., Davidson W.S., He Z., McClelland R.L., Rimm E.B., Jensen M.K. (2020). Protein-Defined Subspecies of HDLs (High-Density Lipoproteins) and Differential Risk of Coronary Heart Disease in 4 Prospective Studies. Arter. Thromb. Vasc. Biol..

[67] Koch M., DeKosky S.T., Goodman M., Sun J., Furtado J.D., Fitzpatrick A.L., Mackey R.H., Cai T., Lopez O.L., Kuller L.H. (2020). Association of Apolipoprotein E in Lipoprotein Subspecies with Risk of Dementia. JAMA Netw. Open.

[68] Sutter I., Park R., Othman A., Rohrer L., Hornemann T., Stoffel M., Devuyst O., von Eckardstein A. (2014). Apolipoprotein M modulates erythrocyte efflux and tubular reabsorption of sphingosine-1-phosphate. J. Lipid Res..

[69] Ruiz M., Frej C., Holmer A., Guo L.J., Tran S., Dahlback B. (2017). High-Density Lipoprotein-Associated Apolipoprotein M Limits Endothelial Inflammation by Delivering Sphingosine-1-Phosphate to the Sphingosine-1-Phosphate Receptor 1. Arter. Thromb. Vasc. Biol..

[70] Liu M., Allegood J., Zhu X., Seo J., Gebre A.K., Boudyguina E., Cheng D., Chuang C.-C., Shelness G.S., Spiegel S. (2015). Uncleaved ApoM Signal Peptide Is Required for Formation of Large ApoM/Sphingosine 1-Phosphate (S1P)-enriched HDL Particles. J. Biol. Chem..

[71] Kurano M., Tsukamoto K., Shimizu T., Kassai H., Nakao K., Aiba A., Hara M., Yatomi Y. (2020). Protection Against Insulin Resistance by Apolipoprotein M/Sphingosine-1-Phosphate. Diabetes.

[72] Kurano M., Hara M., Tsuneyama K., Sakoda H., Shimizu T., Tsukamoto K., Ikeda H., Yatomi Y. (2014). Induction of insulin secretion by apolipoprotein M, a carrier for sphingosine 1-phosphate. Biochim. Biophys. Acta.

[73] Elsoe S., Ahnstrom J., Christoffersen C., Hoofnagle A.N., Plomgaard P., Heinecke J.W., Binder C.J., Bjorkbacka H., Dahlback B., Nielsen L.B. (2012). Apolipoprotein M binds oxidized phospholipids and increases the antioxidant effect of HDL. Atherosclerosis.

[74] Christensen P.M., Bosteen M.H., Hajny S., Nielsen L.B., Christoffersen C. (2017). Apolipoprotein M mediates sphingosine-1-phosphate efflux from erythrocytes. Sci. Rep..

[75] Ahnstrom J., Axler O., Dahlback B. (2010). HDL Stimulates apoM Secretion. Protein Pept. Lett..

[76] Hong B.V., Zheng J., Agus J.K., Tang X., Lebrilla C.B., Jin L.W., Maezawa I., Erickson K., Harvey D.J., DeCarli C.S. (2022). High-Density Lipoprotein Changes in Alzheimer’s Disease Are APOE Genotype-Specific. Biomedicines.

[77] Robert J., Button E.B., Stukas S., Boyce G.K., Gibbs E., Cowan C.M., Gilmour M., Cheng W.H., Soo S.K., Yuen B. (2017). High-density lipoproteins suppress Abeta-induced PBMC adhesion to human endothelial cells in bioengineered vessels and inmonoculture. Mol. Neurodegener..

[78] Robert J., Button E.B., Yuen B., Gilmour M., Kang K., Bahrabadi A., Stukas S., Zhao W., Kulic I., Wellington C.L. (2017). Clearance ofbeta-amyloid is facilitated by apolipoprotein Eand circulating high-density lipoproteins in bioengineered human vessels. eLife.

[79] Robert J., Button E.B., Martin E.M., McAlary L., Gidden Z., Gilmour M., Boyce G., Caffrey T.M., Agbay A., Clark A. (2020). Cerebrovascular amyloid Angiopathy in bioengineered vessels is reduced by high-density lipoprotein particles enriched in Apolipoprotein E. Mol. Neurodegener..

[80] Malajczuk C.J., Mancera R.L. (2025). Molecular Simulation of the Binding of Amyloid Beta to Apolipoprotein A-I in High-Density Lipoproteins. Int. J. Mol. Sci..

[81] Koudinov A.R., Berezov T.T., Kumar A., Koudinova N.V. (1998). Alzheimer’s amyloid beta interaction with normal human plasma high density lipoprotein: Association with apolipoprotein and lipids. Clin. Chim. Acta.

[82] Kakava S., Melo M.J.G., Cheng W.H., Gaudio I.D., Viertl D., Bernhard S., Schlumpf E., Croyal M., Voloviceva E., Fan J. (2025). Both the low-density lipoprotein receptor and apolipoprotein E define blood-borne high-density lipoprotein entry into the brain. bioRxiv.

[83] Fung K.Y., Wang C., Nyegaard S., Heit B., Fairn G.D., Lee W.L. (2017). SR-BI Mediated Transcytosis of HDL in Brain Microvascular Endothelial Cells Is Independent of Caveolin, Clathrin, and PDZK1. Front. Physiol..

[84] Thanopoulou K., Fragkouli A., Stylianopoulou F., Georgopoulos S. (2010). Scavenger receptor class B type I (SR-BI) regulates perivascular macrophages and modifies amyloid pathology in an Alzheimer mouse model. Proc. Natl. Acad. Sci. USA.

[85] Lu N., Moran-Losada P., Hahn P., Saksena A., Tapp E., Chadarevian J.P., Dong W., Shi S.M., Shuken S.R., Guldner I. (2024). Circulatory proteins shape microglia state and boost phagocytosis. bioRxiv.

[86] Simmons C.R., Zou F., Younkin S.G., Estus S. (2011). Evaluation of the global association between cholesterol-associated polymorphisms and Alzheimer’s disease suggests a role for rs3846662 and HMGCR splicing in disease risk. Mol. Neurodegener..

[87] Chang X.L., Tan L., Tan M.S., Wang H.F., Tan C.C., Zhang W., Zheng Z.J., Kong L.L., Wang Z.X., Jiang T. (2016). Association of HMGCR polymorphism with late-onset Alzheimer’s disease in Han Chinese. Oncotarget.

[88] Leduc V., De Beaumont L., Theroux L., Dea D., Aisen P., Petersen R.C., Dufour R., Poirier J., the Alzheimer’s Disease Neuroimaging Initiative (2015). HMGCR is a genetic modifier for risk, age of onset and MCI conversion to Alzheimer’s disease in a three cohorts study. Mol. Psychiatry.

[89] Williams D.M., Finan C., Schmidt A.F., Burgess S., Hingorani A.D. (2020). Lipid lowering and Alzheimer disease risk: A mendelian randomization study. Ann. Neurol..

[90] Benn M., Nordestgaard B.G., Frikke-Schmidt R., Tybjaerg-Hansen A. (2017). Low LDL cholesterol, PCSK9 and HMGCR genetic variation, and risk of Alzheimer’s disease and Parkinson’s disease: Mendelian randomisation study. BMJ.

[91] Nordestgaard L.T., Hanson A., Sanderson E., Anderson E., Walker V., Tybjaerg-Hansen A., Smith G.D., Nordestgaard B.G. (2025). Cholesterol-lowering drug targets reduce risk of dementia: Mendelian randomization and meta-analyses of 1 million individuals. Alzheimer’s Dement..

[92] Olmastroni E., Molari G., De Beni N., Colpani O., Galimberti F., Gazzotti M., Zambon A., Catapano A.L., Casula M. (2022). Statin use and risk of dementia or Alzheimer’s disease: A systematic review and meta-analysis of observational studies. Eur. J. Prev. Cardiol..

[93] Westphal Filho F.L., Moss Lopes P.R., Menegaz de Almeida A., Sano V.K.T., Tamashiro F.M., Goncalves O.R., de Moraes F.C.A., Kreuz M., Kelly F.A., Silveira Feitoza P.V. (2025). Statin use and dementia risk: A systematic review and updated meta-analysis. Alzheimer’s Dement..

[94] Reddin C., Stankard A., Chan K.Y., Krewer F., Judge C., Canavan M., Davis D.H.J., O’Donnell M. (2025). Association of lipid-lowering therapy with dementia and cognitive outcomes: A systematic review and meta-analysis. Age Ageing.

[95] Oveisgharan S., Yu L., Barnes L.L., Agrawal S., Schneider J.A., Bennett D.A., Buchman A.S. (2022). Association of Statins with Cerebral Atherosclerosis and Incident Parkinsonism in Older Adults. Neurology.

[96] Palermo G., Giannoni S., Giuntini M., Belli E., Frosini D., Siciliano G., Ceravolo R. (2021). Statins in Parkinson’s Disease: Influence on Motor Progression. J. Park. Dis..

[97] Ke L., Chen Z., Fan F., Zou X., Yi L., Zuo H., Cheng O. (2026). Association of Statin use with Parkinson’s Disease Progression in a Prospective Cohort Study and Multi-omics Analyses. J. Mol. Neurosci..

[98] Zhang L., Zhang L., Zhao J., Zhang W., Zhang H. (2025). Proprotein Convertase Subtilisin/Kexin Type 9 (PCSK9) in Alzheimer’s Disease: Recent Advances and Controversies. Mol. Neurobiol..

[99] Miao J., Wang J., Zhou W., Guo J. (2026). Small-molecule PCSK9 inhibition enhances BBB amyloid-beta clearance and suppresses microglial inflammation in Alzheimer’s disease models. Sci. Rep..

[100] Postmus I., Trompet S., de Craen A.J., Buckley B.M., Ford I., Stott D.J., Sattar N., Slagboom P.E., Westendorp R.G., Jukema J.W. (2013). PCSK9 SNP rs11591147 is associated with low cholesterol levels but not with cognitive performance or noncardiovascular clinical events in an elderly population. J. Lipid Res..

[101] Tao Q., Ang T.F.A., Huang J., Itchapurapu I.S., Mez J., Alosco M., Au R., Farrer L.A., Zhang X., Qiu W.Q. (2026). Blood PCSK9 Impacts Alzheimer’s Disease Risk in an APOE Genotype-Dependent Manner: A Prospective Cohort Study. Health Sci. Rep..

[102] Huang Q., Zhang Q., Cao B. (2024). Causal relationship between PCSK9 inhibitor and common neurodegenerative diseases: A drug target Mendelian randomization study. Brain Behav..

[103] Sabatine M.S., Giugliano R.P., Keech A.C., Honarpour N., Wiviott S.D., Murphy S.A., Kuder J.F., Wang H., Liu T., Wasserman S.M. (2017). Evolocumab and Clinical Outcomes in Patients with Cardiovascular Disease. N. Engl. J. Med..

[104] Seijas-Amigo J., Mauriz-Montero M.J., Suarez-Artime P., Gayoso-Rey M., Estany-Gestal A., Casas-Martinez A., Gonzalez-Freire L., Rodriguez-Vazquez A., Perez-Rodriguez N., Villaverde-Pineiro L. (2023). Cognitive Function with PCSK9 Inhibitors: A 24-Month Follow-Up Observational Prospective Study in the Real World-MEMOGAL Study. Am. J. Cardiovasc. Drugs.

[105] Tall A.R., Rader D.J. (2018). Trials and Tribulations of CETP Inhibitors. Circ. Res..

[106] Poliakova T., Wellington C.L. (2023). Roles of peripheral lipoproteins and cholesteryl ester transfer protein in the vascular contributions to cognitive impairment and dementia. Mol. Neurodegener..

[107] Schmidt A.F., Davidson M.H., Ditmarsch M., Kastelein J.J., Finan C. (2024). Lower activity of cholesteryl ester transfer protein (CETP) and the risk of dementia: A Mendelian randomization analysis. Alzheimer’s Res. Ther..

[108] Phénix J., Sarty I., Katz M.S., Nie H., Kerksiek A., Kiss R.S., Lütjohann D., Pastor W.A., Poirier J., Munter L.M. (2024). Reducing CETP activity prevents memory decline in an Alzheimer’s disease mouse model. bioRxiv.

[109] Davidson M.H., Szarek M., Scheltens P., Vijverberg E., Hsieh A., Ditmarsch M., Kling D., Curcio D., Nicholls S.J., Ray K.K. (2026). Effect of obicetrapib, a potent cholesteryl ester transfer protein inhibitor, on p-tau217 levels in patients with cardiovascular disease. J. Prev. Alzheimer’s Dis..

[110] Gaudet D., Gipe D.A., Pordy R., Ahmad Z., Cuchel M., Shah P.K., Chyu K.Y., Sasiela W.J., Chan K.C., Brisson D. (2017). ANGPTL3 Inhibition in Homozygous Familial Hypercholesterolemia. N. Engl. J. Med..

[111] Gagnon E., Gill D., Chabot D., Cronje H.T., Yuan S., Brennan S., Theriault S., Burgess S., Arsenault B.J., Dib M.J. (2025). Evaluating the Cardiometabolic Efficacy and Safety of Lipoprotein Lipase Pathway Targets in Combination with Approved Lipid-Lowering Targets: A Drug Target Mendelian Randomization Study. Circ. Genom. Precis. Med..

[112] Zhang H., Zhou Z., Gu J., Lin Y., Yan Y., Chen X., Fan M., Huang Y. (2025). Genetic insights of lipid metabolism and lipid-lowering drugs with Lewy body dementia risk: Evidence from Mendelian randomization. Prog. Neuropsychopharmacol. Biol. Psychiatry.

[113] Li H., Wei J., Zheng Z., Wang R., Qu M., Liu J., Lu G., Li X., Gong W. (2025). The therapeutic potential of recombinant ANGPTL4 in Parkinson’s disease: Evidence from in vivo and in vitro studies. Free Radic. Biol. Med..

[114] Tsimikas S. (2025). Anti-apoC-III Therapies and Implications for Treatment of Pancreatitis and Cardiovascular Disease. Curr. Atheroscler. Rep..

[115] Glittenberg M., Flaten Z., Kumar S.K.S.R., Li D. (2023). Apolipoprotein C proteins in plasma non-high-density lipoprotein fractions and development of Alzheimer’s disease, central arterial stiffness, and cerebral small vessel disease in older adults. Alzheimer’s Dement..

[116] Morofuji Y., Nakagawa S., Ujifuku K., Fujimoto T., Otsuka K., Niwa M., Tsutsumi K. (2022). Beyond Lipid-Lowering: Effects of Statins on Cardiovascular and Cerebrovascular Diseases and Cancer. Pharmaceuticals.

[117] Holzer M., Trieb M., Konya V., Wadsack C., Heinemann A., Marsche G. (2013). Aging affects high-density lipoprotein composition and function. Biochim. Biophys. Acta.

[118] Savulescu-Fiedler I., Dorobantu-Lungu L.R., Dragosloveanu S., Benea S.N., Dragosloveanu C.D.M., Caruntu A., Scheau A.E., Caruntu C., Scheau C. (2025). The Cross-Talk Between the Peripheral and Brain Cholesterol Metabolisms. Curr. Issues Mol. Biol..

[119] Camacho J., Moliné T., Bonaterra-Pastra A., Ramón y Cajal S., Martínez-Sáez E., Hernández-Guillamon M. (2019). Brain ApoA-I, ApoJ and ApoE Immunodetection in Cerebral Amyloid Angiopathy. Front. Neurol..

